# Socioeconomic position, lifestyle habits and biomarkers of epigenetic aging: a multi-cohort analysis

**DOI:** 10.18632/aging.101900

**Published:** 2019-04-14

**Authors:** Giovanni Fiorito, Cathal McCrory, Oliver Robinson, Cristian Carmeli, Carolina Ochoa Rosales, Yan Zhang, Elena Colicino, Pierre-Antoine Dugué, Fanny Artaud, Gareth J McKay, Ayoung Jeong, Pashupati P Mishra, Therese H Nøst, Vittorio Krogh, Salvatore Panico, Carlotta Sacerdote, Rosario Tumino, Domenico Palli, Giuseppe Matullo, Simonetta Guarrera, Martina Gandini, Murielle Bochud, Emmanouil Dermitzakis, Taulant Muka, Joel Schwartz, Pantel S Vokonas, Allan Just, Allison M Hodge, Graham G Giles, Melissa C Southey, Mikko A Hurme, Ian Young, Amy Jayne McKnight, Sonja Kunze, Melanie Waldenberger, Annette Peters, Lars Schwettmann, Eiliv Lund, Andrea Baccarelli, Roger L Milne, Rose A Kenny, Alexis Elbaz, Hermann Brenner, Frank Kee, Trudy Voortman, Nicole Probst-Hensch, Terho Lehtimäki, Paul Elliot, Silvia Stringhini, Paolo Vineis, Silvia Polidoro, the BIOS Consortium, the Lifepath consortium

**Affiliations:** 1 Italian Institute for Genomic Medicine (IIGM), Turin, Italy; 2 The Irish Longitudinal Study on Ageing, Trinity College Dublin, Dublin, Ireland; 3 MRC-PHE Centre for Environment and Health, Imperial College London, London, UK; 4 Institute of Social and Preventive Medicine, Lausanne University Hospital (CHUV), Lausanne, Switzerland; 5 Department of Epidemiology, Erasmus University Medical Center, Rotterdam, the Netherlands; 6 Centro de Vida Saludable de la Universidad de Concepción, Concepción, Chile; 7 Division of Clinical Epidemiology and Aging Research, German Cancer Research Center (DKFZ), Heidelberg, Germany; 8 Icahn School of Medicine, Mount Sinai, New York, NY 10029, USA; 9 Cancer Epidemiology Division, Cancer Council Victoria, Melbourne, Australia; 10 Centre for Epidemiology and Biostatistics, School of Population and Global Health, The University of Melbourne, Victoria, Australia; 11 Precision Medicine, School of Clinical Sciences at Monash Health, Monash University, Clayton, Victoria, Australia; 12 CESP, Faculté de Médecine - Université Paris-Sud, Faculté de Médecine, UVSQ, Institut National de la Santé et de la Recherche Médicale, Université Paris-Saclay, France; 13 Centre for Public Health, Queen’s University Belfast, Belfast, Northern Ireland; 14 Swiss Tropical and Public Health Institute, Basel, Switzerland; 15 University of Basel, Basel, Switzerland; 16 Department of Clinical Chemistry, Fimlab Laboratories, and Finnish Cardiovascular Research Center - Tampere, Faculty of Medicine and Health Technology, Tampere University, Tampere 33520, Finland; 17 Department of Community Medicine, Faculty of Health Sciences, UiT-The Arctic University of Norway, Tromsø, Norway; 18 NILU Norwegian Institute for Air Research, The Fram Centre, Tromsø, Norway; 19 Fondazione IRCCS - Istituto Nazionale dei Tumori, Milan, Italy; 20 Department of Clinical Medicine and Surgery, University of Naples Federico II, Naples, Italy; 21 Piedmont Reference Centre for Epidemiology and Cancer Prevention (CPO Piemonte), Turin, Italy; 22 Cancer Registry and Histopathology Department, 'Civic - M. P. Arezzo' Hospital, ASP Ragusa, Ragusa, Italy; 23 Istituto per lo Studio, la Prevenzione e la Rete Oncologica (ISPRO Toscana), Florence, Italy; 24 Department of Medical Sciences, University of Torino, Torino, Italy; 25 Environmental Epidemiological Unit, Regional Environmental Protection Agency, Piedmont Region, Torino, Italy; 26 Institute of Social and Preventive Medicine, University of Bern, Bern, Switzerland; 27 Department of Environmental Health and Department of Epidemiology, Harvard T.H. School of Public Health, Boston, MA 02115, USA; 28 Department of Epidemiology, Boston University School of Public Health, Boston, MA 02115, USA; 29 Department of Clinical Pathology, The University of Melbourne, Melbourne, Australia; 30 Department of Microbiology and Immunology, Faculty of Medicine and Health Technology, Tampere University, Tampere 33014, Finland; 31 Research Unit of Molecular Epidemiology, Helmholtz Zentrum München, German Research Center for Environmental Health, Neuherberg, Germany; 32 Institute of Epidemiology, Helmholtz Zentrum München, German Research Center for Environmental Health, Neuherberg, Germany; 33 German Center for Cardiovascular Research (DZHK), Munich, Germany; 34 Ludwig-Maximilians-Universität München, Munich, Germany; 35 Institute of Health Economics and Health Care Management, Helmholtz Zentrum München, German Research Center for Environmental Health (GmbH), D-85764, Neuherberg, Germany; 36 Department of Economics, Martin Luther University Halle-Wittenberg, Halle, Germany; 37 Department of Environmental Health Sciences, Columbia University Mailman School of Public Health, New York, NY 10032, USA; 38 Network Aging Research, University of Heidelberg, Heidelberg, Germany; 39 Unit of Population Epidemiology, Primary Care Division, Geneva University Hospitals, Geneva, Switzerland; 40 Equal contribution; 41 Equal senior researcher; 42 See ACKNOWLEDGMENTS AND FUNDING

**Keywords:** socioeconomic position, education, biological aging, epigenetic clocks

## Abstract

Differences in health status by socioeconomic position (SEP) tend to be more evident at older ages, suggesting the involvement of a biological mechanism responsive to the accumulation of deleterious exposures across the lifespan. DNA methylation (DNAm) has been proposed as a biomarker of biological aging that conserves memory of endogenous and exogenous stress during life.

We examined the association of education level, as an indicator of SEP, and lifestyle-related variables with four biomarkers of age-dependent DNAm dysregulation: the total number of stochastic epigenetic mutations (SEMs) and three epigenetic clocks (Horvath, Hannum and Levine), in 18 cohorts spanning 12 countries.

The four biological aging biomarkers were associated with education and different sets of risk factors independently, and the magnitude of the effects differed depending on the biomarker and the predictor. On average, the effect of low education on epigenetic aging was comparable with those of other lifestyle-related risk factors (obesity, alcohol intake), with the exception of smoking, which had a significantly stronger effect.

Our study shows that low education is an independent predictor of accelerated biological (epigenetic) aging and that epigenetic clocks appear to be good candidates for disentangling the biological pathways underlying social inequalities in healthy aging and longevity.

## Introduction

Aging is characterized by a gradual and constant increase in health inequalities across socioeconomic groups [1,2], an association based on strong epidemiological evidence known as the ‘social gradient in health’. On average, individuals with lower socioeconomic position (SEP) have lower life expectancy, higher risk of age-related diseases, and poorer quality of life at older ages compared with less disadvantaged groups. Although lifestyles differ by SEP, unhealthy habits only partially explain this association [3].

The role of epigenetic mechanisms in response to trauma, and evidence for their involvement in intergenerational transmission of biological impacts of traumatic stress have been proposed to explain how social adversity gets biologically embedded [4], leading to differences in biological functionalities among individuals in different social conditions, especially at older ages. Epigenetics, specifically DNA methylation (DNAm) has been proposed as one of the most powerful biomarkers of biological aging and as one of the plausible biological mechanisms by which social adversities get ‘under the skin’ and affect physiological and cellular pathways leading to disease susceptibility [5–7].

Two different mechanisms have been proposed to contribute to age-related DNAm changes: ‘epigenetic drift’ and the ‘epigenetic clock’ that sometimes are used as synonyms even though describe different molecular mechanisms [8–10]. Although both are related to aging, epigenetic drift represents the trend of increasing DNAm variability over time across the whole genome. On the contrary, the epigenetic clock refers to specific CpG sites identified in specific DNA regions at which DNAm levels constantly increase (or decrease depending on the site) during aging and can be used to predict chronological age with high accuracy [11]. Two measures of epigenetic clocks have gained considerable popularity, Horvath [11] and Hannum [12], and the concept of epigenetic aging acceleration (EAA) has been introduced as the difference between predicted DNAm age and chronological age. EAA has been associated with all-cause mortality, cancer incidence and neurodegenerative disorders, as well as non-communicable disease risk factors such as obesity, poor physical activity, unhealthy diet, cumulative lifetime stress and infections [13,14]. Recently, Levine and colleagues introduced a ‘next-generation epigenetic clock’ that is based on a set of CpGs associated with a complex set of clinical measures thought to assess the ‘phenotypic age’ [15]. Levine EAA was found to outperform other measures with regard to the prediction of a variety of aging outcomes, including all-cause mortality, the incidence of and survival from cancer, and physical functioning [15].

In contrast to EAA, epigenetic drift is a mechanism that involves the whole-genome, where age-related genomic instability and chromatin deterioration lead to increased variability of genome-wide DNAm levels at older ages [16]. Different statistical approaches can be used to evaluate the impact of epigenetic drift on aging and disease susceptibility. For example, Teschendorff and colleagues suggested that methods based on differential DNAm variability could identify risk markers more robustly than statistical measures based on differences in mean DNAm levels [17]. Gentilini and colleagues developed an analytical approach to identify these stochastic epimutations (SEMs) [18] from genome-wide DNAm data, showing that the number of SEMs increases exponentially with age although there is high variability within individuals of the same age. A higher number of SEMs was found to be associated with X chromosome inactivation skewing in women (an age-related condition and risk factor for cancer), hepatocellular carcinoma tumor staging [18,19], and unhealthy exposure such as cigarette smoking, alcohol intake [20] and exposure to toxicants [21], suggesting SEMs as possible biomarkers of exposure-related accumulation of DNA damage during lifespan.

Given the above, it can be assumed that the various epigenetic clocks (Horvath’s, Hannum’s, Levine’s) and the total number of SEMs describe different aspects of the biological (epigenetic) aging process. We previously showed a dose-response relationship between SEP and EAA. Further, our results suggest that the effect could be partially reversible by improving social conditions during life [5]. In addition, ours and two more recent studies indicate that childhood SEP might have a stronger effect on EAA than adulthood SEP [22,23].

Despite extensive research in the field, to date no studies have compared the effect of SEP on epigenetic aging biomarkers with those of other lifestyle-related risk factors for age-related diseases. We aimed to systematically investigate the association of education level, as a proxy for SEP, with the total number of SEMs and ‘accelerated aging’ as assessed using the three epigenetic clocks, and to compare the independent effect of low education with those of the main modifiable risk factors for premature aging: smoking, obesity, alcohol intake and physical inactivity, by conducting a meta-analysis including data for more than 16,000 individuals belonging to 18 cohort studies from 12 different countries worldwide.

## RESULTS

After quality control and sample filtering, we analyzed blood DNAm data from 16,245 individuals from 18 cohort studies. The main characteristics of the study sample are shown in Table 1. For each epigenetic outcome, we report the results of a meta-analysis of the association with education, smoking, obesity, alcohol intake and physical activity in Table 2. Model 1 includes age, sex, and cohort-specific covariates as adjustment variables whereas Model 2 is the fully adjusted model (additionally adjusted for smoking, BMI, alcohol intake and physical activity).

**Table 1:** Study sample descriptive statistics.

**Cohort short name**	**Cohort full name**	**Country**	**Illumina BeadChip**	**N**	**Mean age (min - max)**	**Female N(%)**	**Reference**
AIRWAVE	The Airwave Health Monitoring Study	UK	Illumina EPIC chip (850K)	1,127	41 (13 - 65)	458 (41%)	[46]
EXPOsOMICS 'EPIC CVD'	The European Prospective Investigation into Cancer and Nutrition - EXPOsOMICS subsample	Italy	Illumina 450K BeadChip	313	57 (35 - 75)	167 (53%)	[47]
EPIC	The European Prospective Investigation into Cancer and Nutrition	Italy	Illumina 450K BeadChip	1,803	53 (35 - 75)	1,114 (62%)	[48]
ESTHER 1	Epidemiological investigations on chances of preventing, recognizing early and optimally treating chronic diseases in an elderly population	Germany	Illumina 450K BeadChip	1,000	62 (48 - 75)	500 (50%)	[49]
ESTHER 2	Epidemiological investigations on chances of preventing, recognizing early and optimally treating chronic diseases in an elderly population	Germany	Illumina EPIC chip (850K)	864	63 (48 - 75)	390 (45%)	[49]
KORA	Cooperative Health Research in the Region of Augsburg (KORA-F4)	Germany	Illumina 450K BeadChip	1,727	61 (32 - 81)	882 (51%)	[50]
MCCS	Melbourne Collaborative Cohort Study	Australia	Illumina 450K BeadChip	2,817	59 (40 - 70)	1,095 (39%)	[51]
NAS	Normative aging study	USA	Illumina 450K BeadChip	624	72 (55 - 91)	0 (0%)	[52]
NOWAC	The Norwegian Women and Cancer Study	Norway	Illumina 450K BeadChip	632	56 (47 - 63)	632 (100%)	
NICOLA	Northern Ireland Cohort Longitudinal Study of Ageing	Northern Ireland	Illumina EPIC chip (850K)	1,929	64 (40 - 96)	988 (51%)	[53]
RS-Bios	Rotterdam Study 1,2	Netherlands	Illumina 450K BeadChip	720	68 (52 - 80)	304 (42%)	[54]
RSIII-1	Rotterdam Study 3	Netherlands	Illumina 450K BeadChip	730	60 (46 - 89)	335 (46%)	[54]
SAPALDIA	Swiss Study on Air Pollution and Lung Diseases in Adults	Switzerland	Illumina 450K BeadChip	402	57 (38 - 81)	184 (46%)	[REMOVED HYPERLINK FIELD] [55]
SKIPOGH a	Swiss Kidney Project on Genes in Hypertension	Switzerland	Illumina 450K BeadChip	250	51 (26 - 82)	132 (53%)	[56]
SKIPOGH b	Swiss Kidney Project on Genes in Hypertension	Switzerland	Illumina EPIC chip (850K)	451	54 (25 - 89)	231 (51%)	[56]
TERRE	Case-control study of Parkinson’s disease in French farmers (only controls were used)	France	Illumina EPIC chip (850K)	174	67 (41 - 76)	80 (46%)	[57]
TILDA	The Irish Longitudinal Study on aging	Ireland	Illumina EPIC chip (850K)	490	62 (50 - 80)	246 (50%)	[58]
YFS	Young Finns Study	Finland	Illumina 450K BeadChip	186	44 (34 - 49)	72 (39%)	[59]

**Table 2:** Results of linear regressions using epigenetic aging biomarkers as outcomes and lifestyle related risk factors as predictors.

		**SEMs**		**HorvathEAA**	
		**Model 1**	**Model 2**	**Model 1**	**Model 2**
**Education (ref: High)**	Medium	0.23 (0.02; 0.44)*	0.17 (-0.07; 0.42)	0.11 (-0.07; 0.28)	0.11 (-0.08; 0.29)
	Low	0.34 (0.11; 0.58)**	0.28 (0.04; 0.51)*	0.22 (0.03; 0.41)*	**0.19 (0.00; 0.39)^+^**
**Smoking (ref: Never)**	Former	0.32 (0.14; 0.49)***	0.33 (0.16; 0.51)***	0.13 (-0.04; 0.29)	0.11 (-0.05; 0.26)
	Current	0.53 (0.32; 0.73)***	0.51 (0.30; 0.72)***	-0.06 (-0.24; 0.13)	-0.08 (-0.27; 0.12)
**Obesity (ref: BMI < 25)**	BMI < 30	-0.01 (-0.18; 0.16)	-0.01 (-0.18; 0.15)	0.37 (0.22; 0.52)***	0.33 (0.18; 0.48)***
	BMI ≥ 30	-0.06 (-0.26; 0.15)	-0.07 (-0.27; 0.14)	0.45 (0.27; 0.63)***	0.43 (0.24; 0.61)***
**Alcohol (ref: Abstainer)**	Occasional	-0.12 (-0.31; 0.08)	-0.10 (-0.29; 0.08)	-0.02 (-0.19; 0.15)	0.00 (-0.18; 0.18)
	Habitual	0.22 (-0.05; 0.49)	0.15 (-0.11; 0.4)	0.19 (-0.07; 0.44)	0.25 (0.00; 0.49)*
**Physical activity (ref: High)**	Medium	0.00 (-0.21; 0.21)	-0.03 (-0.21; 0.15)	0.05 (-0.11; 0.21)	0.08 (-0.09; 0.24)
	Low	0.03 (-0.28; 0.35)	-0.03 (-0.32; 0.26)	0.22 (0.05; 0.39)*	0.22 (0.04; 0.40)*
		**HannumEAA**		**LevineEAA**	
		**Model 1**	**Model 2**	**Model 1**	**Model 2**
**Education (ref: High)**	Medium	0.32 (0.14; 0.49)***	0.27 (0.08; 0.46)**	0.50 (0.22; 0.79)***	**0.31 (-0.05; 0.67)^+^**
	Low	0.34 (0.17; 0.52)***	0.31 (0.14; 0.48)***	0.84 (0.50; 1.17)***	0.60 (0.25; 0.94)***
**Smoking (ref: Never)**	Former	0.04 (-0.08; 0.16)	0.01 (-0.12; 0.13)	0.60 (0.37; 0.84)***	0.52 (0.28; 0.77)***
	Current	0.24 (0.06; 0.42)**	0.17 (0.00; 0.35)*	1.57 (1.31; 1.82)***	1.41 (1.14; 1.67)***
**Obesity (ref: BMI < 25)**	BMI < 30	0.17 (0.05; 0.28)**	0.15 (0.03; 0.27)*	0.37 (0.13; 0.62)**	0.33 (0.11; 0.55)**
	BMI ≥ 30	0.22 (0.07; 0.36)**	0.20 (0.05; 0.34)*	1.08 (0.79; 1.37)***	1.01 (0.74; 1.28)***
**Alcohol (ref: Abstainer)**	Occasional	-0.05 (-0.19; 0.09)	0.03 (-0.11; 0.17)	-0.08 (-0.36; 0.20)	0.10 (-0.14; 0.34)
	Habitual	0.14 (-0.03; 0.31)	0.21 (0.04; 0.39)*	0.88 (0.49; 1.26)***	0.91 (0.57; 1.25)***
**Physical activity (ref: High)**	Medium	0.07 (-0.08; 0.22)	0.07 (-0.07; 0.20)	0.16 (-0.17; 0.49)	0.20 (-0.04; 0.44)
	Low	0.08 (-0.15; 0.32)	0.05 (-0.20; 0.30)	0.42 (-0.12; 0.96)	0.31 (-0.13; 0.74)

*** p < 0.001; ** p < 0.01; * p < 0.05; + p < 0.10

Model 1 includes age, sex, and cohort specific covariates; Model 2 includes additional adjustment for education, smoking, BMI, alcohol and physical activity.

For the three epigenetic clocks, the estimated differences presented in Table 2 (βs) represent the change in biological age (in years) compared with the reference group. Accordingly, the estimated effects of risk factors on SEMs were re-scaled to be expressed in years as for the three epigenetic clocks using a two-step approach based on the Cohen’s D statistic, described in the supplementary text.

***Education:*** The level of education was significantly associated with the four biomarkers investigated. In Model 1 (minimally adjusted), lower educated individuals had a higher number of SEMs β = 0.34 (95% CI 0.11; 0.58), higher Horvath EAA β = 0.22 (0.03; 0.41), higher Hannum EAA β = 0.34 (0.17; 0.52), and higher Levine EAA β = 0.84 (0.50; 1.17), compared with the higher educated group who constituted the reference category. The observed associations were still significant after the inclusion of smoking, BMI, alcohol and physical activity in the regression models (Model 2), but the estimated effects were moderately reduced. Comparing the two extreme categories (low vs. high education) the estimated effects were: SEMs β = 0.28 (0.04; 0.51), Horvath EAA β = 0.19 (0.00; 0.39), Hannum EAA β = 0.31 (0.14; 0.48), and Levine EAA β = 0.60 (0.25; 0.94) in the full multivariable adjusted models. Interestingly, the intermediate education group ranked between the high and low education group supporting a dose-response effect (Table 2).

***Other modifiable risk factors:*** Current smokers had a higher number of SEMs, higher Hannum EAA and higher Levine EAA compared with never smokers. The estimated effects were slightly reduced in Model 2 compared with Model 1 when adjusted additionally for other covariates. Further, former smokers had intermediate outcomes between never and current smokers (Table 2). The estimated effect size of the association between smoking and epigenetic aging biomarkers was comparable to those observed for education, except for the magnitude of the association with Levine EAA, which was significantly higher: β = 1.57 (1.31; 1.82) in Model 1; β = 1.41 (1.14; 1.67) in Model 2. A similar pattern of associations was observed looking at the effects of obesity on epigenetic aging biomarkers. Obese individuals (BMI ≥ 30) had higher Horvath EAA, higher Hannum EAA, and higher Levine EAA. As previously described for education and smoking, the effects estimated in Model 2 were slightly lower compared with Model 1, and a dose-response association was observed. The estimated effects of obesity were comparable to those of education except for Levine EAA, which was significantly higher: β = 1.08 (0.79; 1.37) in Model 1; β = 1.01 (0.74; 1.28) in Model 2. Looking at alcohol intake, we did not observe any significant difference comparing abstainers and occasional drinkers, but habitual drinkers had higher Horvath EAA, Hannum EAA and Levine EAA. As observed for the other risk factors, the higher estimated effects were observed for Levine’s indicator: β = 0.88 (0.49; 1.26) in Model 1; β = 0.91 (0.57; 1.25) in Model 2. Finally, low physical activity was associated with higher Horvath EAA in both Model 1 β = 0.22 (0.05; 0.39) and Model 2 β = 0.22 (0.04; 0.40).

**Figure 1:**
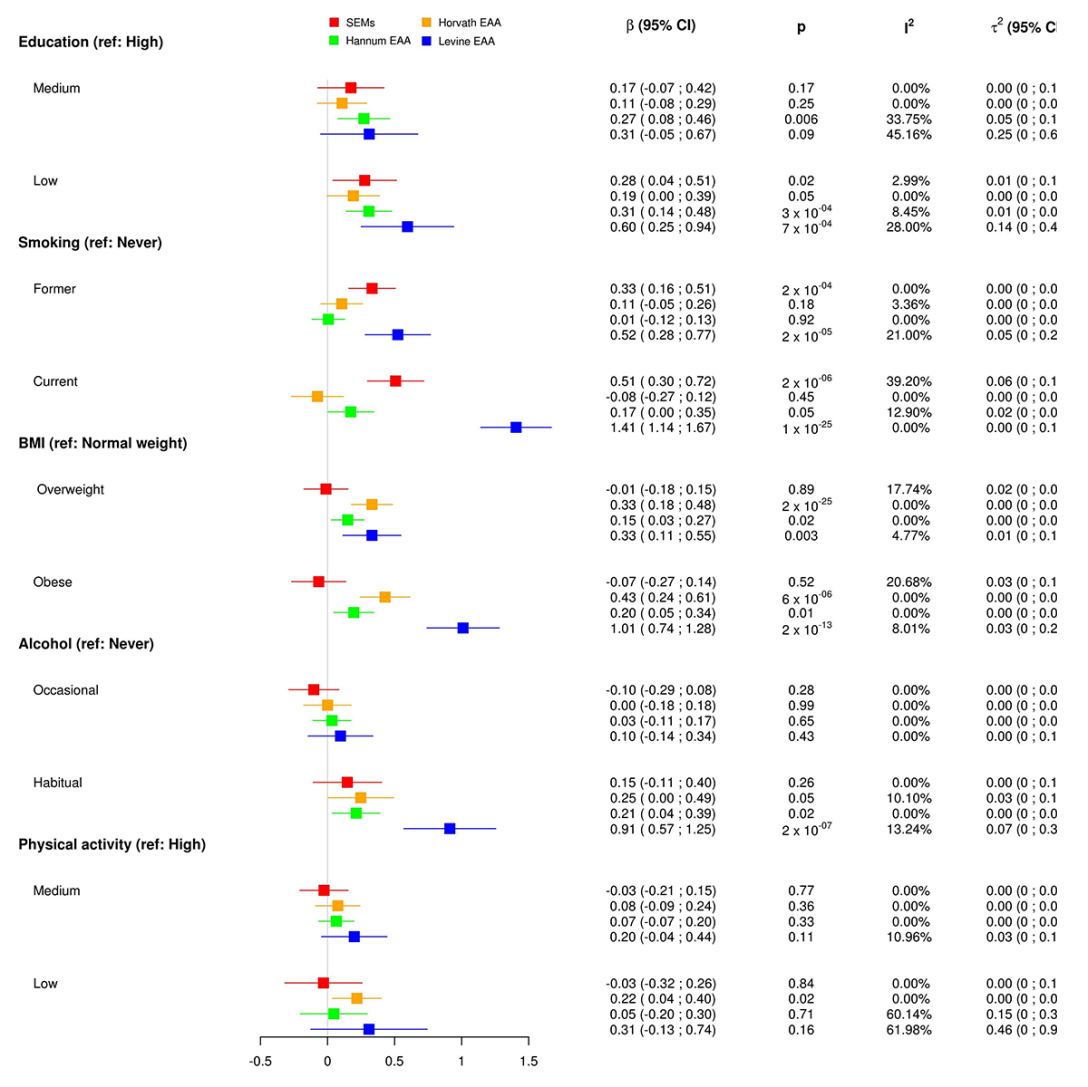
Effect sizes (interpretable as years of increasing/decreasing epigenetic age) of the association between different risk factors and four epigenetic aging biomarkers: total number of stochastic epigenetic mutations (SEMs, red), Horvath epigenetic age acceleration (orange), Hannum epigenetic age acceleration (green) and Levine epigenetic age acceleration next-generation clock (blue).

Figure 1 shows a graphical representation of the results (Model 2) using forest plot which allows one to compare the effect of each risk factor considered in the present paper on the four DNAm outcomes.

In sensitivity analyses, we examined the white blood cell (WBC) adjusted epigenetic aging measures (described in Methods), and found similar associations as the ones described above (Table S1). Further, for each risk factor, we evaluated the interaction with age and sex. Our results indicated no significant differences in associations between men and women, whereas we found a significant interaction with age for the association of SEMs with education, smoking, and obesity, with a significantly stronger effect in older individuals (Table S2).

We examined whether SEMs were randomly distributed across the genome or are enriched in functional genomic regions. We observed overlap between the genomic position of SEMs and regions associated with open chromatin states, and shores (p=0.03, p=0.02 respectively, Table S3). Considering the categories defined by the Encyclopedia of DNA Elements (ENCODE) project with Chromatin ImmunoPrecipitation Sequencing (ChIP-Seq) experiments on human embryonic stem cells (hESC), we found enrichment of SEMs in ‘inactive/poised promoters’ (p<0.0001, Table S4), ‘heterochromatin/low signal/CNV’ (p<0.0001, Table S4), and ‘Polycomb-repressed’ regions (p=0.001, Table S4). Furthermore, we found significant overlap with transcription factor binding sites (TFBSs) targeted by two members of the Polycomb repressive complex-2 (PRC2): EZH2 and SUZ12 (p<0.0001, Table S5).

## DISCUSSION

Social inequalities in health have been extensively reported and accelerated age-dependent DNAm dysregulation has been proposed as one of the biomolecular mechanisms mediating this association [5,24,25]. In this study, we examined the effect on DNAm biomarkers of aging of being in the low education group compared with those of other lifestyle-related risk factors: smoking, obesity, alcohol intake, and low levels of physical activity. We used education as the proxy for SEP as it was the only socioeconomic indicator that was available in all the cohorts and it is usually completed before the onset of many chronic diseases, therefore reducing the risk of reverse causation [26]. Lower educational attainment was associated with EAA according to the ‘first generation’ clocks including Horvath’s and Hannum’s. However, previously it was not clear whether the observed associations depend on other factors associated with low education [6,27]. For example, Karlsson Linnér and colleagues argue that the association of educational attainment and epigenetic aging is mainly mediated by maternal smoking during pregnancy and smoking during adulthood [6]. To clarify this issue and to increase the epidemiological evidence in the field, we have examined four biomarkers of age-dependent DNAm dysregulation: the total number of SEMs and three epigenetic clocks (Horvath, Hannum and Levine).

Although all the biomarkers are related to aging, they did not show the same pattern of associations with risk factors, intermediate traits and diseases [28,29], suggesting that these biological age predictors may reflect different facets of the aging process. The total number of SEMs takes into account whole-genome epigenetic deregulation during aging, a process known as ‘epigenetic drift’, and has been proposed as a biomarker of exposure-related accumulation of DNA damage during the lifespan [20]. It is necessary to clarify that the word ‘epimutation’ is sometimes used in a manner that can be misinterpreted. Although some literature uses this term to refer to epigenetic changes driven by genotype differences, the strict definition of epimutation is a heritable change in gene activity that is not associated with a DNA mutation, but rather, with gain or loss of DNA methylation or other heritable modifications of chromatin [30]. Contrary to the definition of genetic mutations, epimutations are defined as potentially (but not necessarily) reversible changes in gene activity not involving DNA mutations, but rather, gain or loss of DNA methyl groups conserved in cells through mitosis [20,30,31].

In contrast, Horvath’s epigenetic clock is based on DNAm levels at a small subset of CpG sites and is thought to reflect the biological age of different tissues, while Hannum’s epigenetic clock is specific to blood samples. Finally, Levine’s next-generation clock is computed using a subset of CpGs that were associated with several clinical measures representing the health status of an individual and has been proposed as a biomarker of the individual ‘phenotypic age’ [15]. Accordingly, Levine’s measure of age acceleration tends to be more variable than the first-generation clocks (Horvath and Hannum) as evidenced by the finding that the associations based on this marker showed, in general, a higher degree of heterogeneity in the meta-analysis measured with the I^2^ and τ^2^ statistics.

Our results from this meta-analysis of more than 16,000 individuals support our working hypothesis. We found that the four aging biomarkers were associated with different sets of risk factors and that the magnitude of the associations differed depending on the epigenetic aging index. We compared the effects of two nested models: the first minimally adjusted model included age, sex and cohort-specific covariates as adjustments; the second (fully adjusted model) was adjusted for all of the risk factors. We did not observe significant differences comparing estimates from the two models (Table 2), supporting the robustness of the results presented. Interestingly, the effect of low education was independent from the other risk factors examined, as it was significant in both the minimally adjusted and fully adjusted models; and the effect sizes were comparable to that of the other risk factors examined, with the exception of smoking, which had an appreciably larger impact on SEMs and Levine’s measure. Two previous studies from our group that evaluated the association of low SEP with mortality and physical functioning documented strong patterning by SEP [32,33]. The current study provides evidence of the potential role of epigenetic modifications as mediators of the association of low SEP and unhealthy lifestyle habits with adverse outcomes at older ages, and further underscores the importance of considering SEP as an important life course risk factor for premature biological aging.

In sensitivity analyses, we also investigated alternative measures of the epigenetic aging biomarkers corrected for the proportion of WBC (estimated from whole-genome DNAm data). Chen and colleagues refer to the WBC-adjusted epigenetic aging as an ‘intrinsic’ measure of biological aging, which captures cell-intrinsic properties of the aging process, that exhibit some preservation across various cell types and organs [34]. Our results indicate no significant differences in the results using ‘extrinsic’ (non-WBC-adjusted) vs ‘intrinsic’ measures. Similarly, stratified analyses by sex indicated no differential effect between men and women.

Finally, we evaluated the potential differential effects of risk factors by (chronological) age group. We found that the effect of education, smoking and BMI on the total number of SEMs, and the effect of smoking on Hannum EAA was significantly greater for older individuals. These results agree with the ‘epigenetic memory’ hypothesis according to which epigenetic aging biomarkers, particularly SEMs, could reflect the accumulation of deleterious exposures during the lifespan [35].

To elucidate the molecular mechanisms involved in DNAm dysregulation during aging we investigated whether SEMs occurred randomly in the DNA sequence or were enriched in regulatory regions. Our findings confirmed that epimutations preferentially occur in DNA sequences associated with open chromatin (Table S3), as previously observed by Ong et al. [36]. Furthermore, SEMs were enriched in transcriptionally silenced genomic regions such as ‘inactive promoters’, ‘heterochromatin/low signal/copy number variants (CNV)’, and ‘Polycomb-repressed’ regions (Table S4). Specifically, SEMs were more likely to occur in transcription factor binding sites (TFBSs) targeted by two members of Polycomb repressive complex 2 (PRC2): EZH2 and SUZ12 (Table S5). The role of PCR2 proteins and EZH2 specifically in aging and age-related diseases has been extensively investigated in recent years. Targets of PRC2 proteins are enriched for tumor suppressor genes and genes related to mental/neurodegenerative disorders [37–39], providing a link between aging, age-related DNAm modifications and age-related diseases. As an example, downregulations of EZH2 and SUZ12 have been associated with dysregulation of several PRC2 targets including *p53*, a well-known tumor suppressor gene [40]. These results underscore the imperative for further research aimed at identifying disease-specific epimutation signatures.

In conclusion, we have shown that SEP is associated with different biomarkers of biological (epigenetic) aging, which may represent complementary aspects of the aging process. On average, the impact of low education was comparable with that of other lifestyle-related risk factors, with the exception of smoking, for which the effect was more pronounced. Levine’s second-generation clock was more strongly associated with education and the other risk factors (smoking, obesity and alcohol intake) compared with the first-generation epigenetic clocks and epimutation biomarkers. This result is not wholly unexpected because Levine’s indicator was explicitly designed with a two-step procedure to include both CpG sites associated with aging *per se*, and CpG sites predictive of mortality and associated with biomarkers of chronic diseases. Given the above, Levine’s epigenetic clock appeared to be a strong candidate for future studies aiming at disentangling the biological pathways underlying social inequalities in healthy aging and longevity. We confirmed in a very large cross-cohort, cross-country sample, previous observations of ‘accelerated aging’ in individuals of low SEP [6,15]. To the best of our knowledge this is also the first study showing that i) socioeconomic status is associated with the total number of stochastic epimutations, which in turn is a biomarker of adverse health outcomes at older ages; and ii) the impact of low socioeconomic status on age-related epigenetic biomarkers is comparable to that of the major lifestyle-related and modifiable risk factors for age related diseases, with the exception of smoking which had a stronger effect on two out of four epigenetic biomarkers investigated.

This study also has limitations: variable collection and classification were not done in a standardized way for all the cohorts. Similarly, for DNA methylation data, each cohort used its own method for data normalization and batch effect removal. To avoid over-estimation of the effects of education and other risk factors on epigenetic aging biomarkers, we used a REML method that incorporates random study effects around the overall mean, to obtain robust global estimates in meta-analysis.

Education was the only SEP indicator used in this study. Critical alternative SEP indicators like income or occupational position were not available in all of the cohorts used in this meta-analysis. We chose to consider all the variables as categorical, with three levels each. On the one hand, this procedure helped us to compare the effects of low education with that of other risk factors, but on the other hand, could lead to a reduction in statistical power to detect significant associations. The lack of association of epigenetic aging measures with low physical activity is not in line with previous literature [27] which might be explained by heterogeneity in the variable definition across cohorts.

## MATERIALS AND METHODS

### Participating cohorts

We obtained summary-level association statistics from 17 independent cohort studies located in Europe, the United States and Australia (Table 1). The total sample size includes 16,245 individuals of recent European ancestry. All participants provided written informed consent, and all contributing cohorts confirmed compliance with their Local Research Ethics Committees or Institutional Review Boards. Each risk factor was treated as a categorical variable with three categories each; education: ‘High’ (reference), ‘Medium’, ‘Low’; smoking: ‘Never’ (reference), ‘Former’, ‘Current’; obesity: ‘Normal weight (BMI ≤ 25, reference)’, ‘Overweight (25 < BMI ≤ 30)’, ‘Obese (BMI > 30)’; alcohol consumption: ‘Abstainers’ (reference), ‘Occasional drinkers’, ‘Habitual drinkers’; physical activity: ‘High’ (reference), ‘Medium’, ‘Low’ (see the supplementary text for the definition of the categories).

### Identification of stochastic epigenetic mutations

We identified SEMs based on the procedure described by Gentilini et al. [18]. Briefly, for each CpG, considering the distribution of DNAm beta values across all samples, we computed the interquartile range (IQR) - difference between the third quartile (Q3) and the first quartile (Q1) - and we defined a SEM as a methylation value lower than Q1-(3×IQR) or greater than Q3+(3×IQR). Finally, for each individual, we computed the total number of SEMs across all CpGs. Since the total number of SEMs increased exponentially with age, we used a logarithmic transformation of the outcome for all the analyses. In sensitivity analyses, we computed the same measure corrected for potential confounding by differential WBC proportions among individuals. Specifically, for each CpG we calculated the residuals from the regression of DNAm beta values on estimated WBC fractions, and then applied the same procedure described above.

### Computation of epigenetic clock measures

We computed the epigenetic age acceleration (AA) measures according to the algorithm described by Horvath [11]. Briefly, DNAm age was calculated as a weighted average of 353 age-related CpGs (Horvath DNAm age), 71 blood specific age-related CpGs (Hannum DNAm age), and 513 phenotypic age-related CpGs (Levine DNAm age) respectively. Weights were obtained using a penalized regression model (Elastic-net regularization) [11]. Age acceleration (AA) was defined as the difference between epigenetic and chronological age. Since AA may be correlated with chronological age, the ‘extrinsic’ EAA is defined as the residuals of AA on age. For sensitivity analyses, we also computed the ‘intrinsic’ epigenetic age acceleration (IEAA), defined as the residuals from the linear regression of AA on chronological age and WBC percentages [34]. Positive values of EAA (which is by definition independent of age) indicate accelerated aging and negative values decelerated aging.

### Association of epigenetic aging measures with risk factors

We investigated the association of four biological (epigenetic) aging biomarkers with education, smoking, obesity, alcohol and physical activity using each epigenetic measure as the outcome and risk factors as predictors in linear regression models. For each outcome and each risk factor, we ran two regression models. Model 1 was adjusted for age, sex and cohort-specific covariates only (see the supplementary text). Model 2 included additional adjustment for education, smoking, BMI, alcohol intake, physical activity, to derive the mutually adjusted estimate for each risk factor considered in the present paper.

Each cohort provided the results of the analyses as point estimates and 95% confidence intervals based on an R script shared with all the data analysts (available in the Supplementary Materials). The pooled estimated association of risk factors with each outcome were obtained by random effect maximum likelihood (REML) meta-analysis using the R package *metafor* [41]. Heterogeneity among studies was assessed using the I^2^ and τ^2^ statistics.

### SEMs enrichment analysis

The genomic locations of SEMs were annotated by merging the Illumina information on the chromosomal position of each probe with ENCODE/NIH Roadmap ChIP-Seq data for chromatin states and transcription factor binding sites (TFBS) in hESC [42–44]. We investigated whether SEMs were randomly distributed across the genome, or were enriched in functional genomic regions using the procedure implemented in the R package *regioneR* [45]. Briefly, the algorithm is specifically designed to test whether a set of genomic *loci* significantly overlap with a set of genomic regions, and includes a permutation procedure that controls for the type I error rate.

## SUPPLEMENTARY MATERIAL

Supplementary Methods

Supplementary Tables

R script 1

R script 2

R script 3
