# Supplementary material for: Socioeconomic position, lifestyle habits and biomarkers of epigenetic aging: a multi-cohort analysis
Source: Aging (Albany NY). 2019 Apr 14;11(7):2045–70. doi: 10.18632/aging.101900 (PMC6503871; doi:10.18632/aging.101900)
Supplement: Supplementary Methods [file aging-11-101900-s001.pdf]

SUPPLEMENTARY MATERIAL

Please browse the links in Full Text version of this manuscript to see R Scripts.

SUPPLEMENTARY METHODS

Cohort description and variable definition

EPIC - Study participants were drawn from the Italian component of the European Prospective Investigation into Cancer and Nutrition (EPIC) cohort, a large general population cohort consisting of ~520,000 individuals, with standardized lifestyle and personal history questionnaires, measured anthropometric data and blood samples collected for DNA extraction [1]. Socio-economic, dietary and lifestyle-related variables were collected at study enrolment through the use of a validated questionnaires. The highest educational attainment was categorized as follow: ‘low’ = primary school or lower; ‘medium’ = secondary school, ‘high’ = university degree or higher. Smoking was categorized as ‘never’, ‘former’ and ‘current’ smokers based on self-reported information. Alcohol was categorized as ‘abstainer’, ‘occasional’ (less than 28 g/day) and ‘habitual’ drinkers (more than 28 g/day). Physical activity was assessed using the Cambridge Physical Activity Index which combines self-reported occupational activity with time participating in cycling and sports. Participants were divided into 3 categories: ‘low’ (sedentary job and no recreational activity), ‘medium’ (at least one of physical job and less than one hour of recreational activity per day), and ‘high’ (sedentary job with >1 hour of recreational activity per day, standing or physical job with some recreational activity, or a heavy manual job). Height and weight were measured at enrolment with a standardized protocol, and body mass index (BMI) was calculated as the ratio between weight in kg and squared height in meters, treated as categorical variable: normal weight = BMI ≤ 25; overweight = 25 < BMI ≤ 30; obese = BMI ≥ 30

Airwave - The Airwave Health Monitoring Study is an occupational cohort of employees of 28 police forces from across Great Britain. Full details of the cohort and methods are available in Elliott et al [2]. The study started recruitment in 2006 and now contains 53,280 participants. The study received ethical approval from the National Health Service Multi-Site Research Ethics Committee (MREC/13/NW/0588). At the baseline health screening, participants underwent health examination, self-completed a computer questionnaire and blood samples in EDTA tubes for DNA extraction. Blood samples were spun at the health clinic and the biological samples were stored in a Thermoporter (LaminarMedica) and frozen at -80 °C long term storage. Covariates in the analysis were categorised from self-report or clinical data as follows: Education was defined as low (completed GCSEs or equivalent only), medium (completed ‘A’ levels or equivalent only) or high (completed university or higher degree). Alcohol use was classed as non-drinker, occasional drinker (≤ 14 alcohol units/week for women and ≤ 21 alcohol units/week for men) or habitual drinker (> 14 alcohol units/week for women and >21 alcohol units/week for men). Physical activity was defined as low, moderate or high based on the scoring protocol of the International Physical Activity Questionnaire [3]. Smoking was categorized as ‘never’, ‘former’ and ‘current’ smokers based on self-reported information. Height and weight were measured at enrolment with a standardized protocol, and body mass index (BMI) was calculated as the ratio between weight in kg and squared height in meters, treated as categorical variable: normal weight = BMI ≤ 25; overweight = 25 < BMI ≤ 30; obese = BMI ≥ 30

The *ESTHER* study is an ongoing population-based cohort study conducted in the federal state of Saarland, Germany [4]. In brief, 9,949 older adults (50-75 years) were recruited by their general practitioners (GPs) during routine health check-ups (offered every two years to people older than 35 years in the German healthcare system) between 2000 and 2002, and followed up thereafter. During the baseline enrolment, epidemiological data (including socio-demographic characteristics, lifestyle factors, and history of major diseases) were collected via a standardized self-administered questionnaire completed by participants and via additional reports from participants’ GPs, and biological samples (blood, stool, urine) were obtained and stored at −80 °C. Educational levels were defined as low [≤9 years], medium [10-11 years], and high [≥= 12 years]. Smoking behaviours were based on self-reported information and classified according to commonly used criteria. An ever-smoker was defined as a subject who had ever smoked ≥100 cigarettes during his or her lifetime, thus excluding rare occasional smoking. An ever-smoker was classified as a former smoker if he or she had stopped smoking for ≥1 year prior to the study. Body mass index (BMI) were categorized as underweight (<18.5 kg/m^2), normal weight (18.5 to <25.0 kg/m^2), overweight (25.0 to <30.0 kg/m^2), or obese (≥30.0 kg/m^2)]. Physical activity were categorized as inactive (<1 hour/week of physical activity), medium/high (≥2 hour/week of vigorous physical activity or ≥2 hour/week of light physical activity), or low (all others)]. Alcohol was categorized as abstainer (0 gram/day), occasional drinker (≤28 gram/day), and habitual drinkers (>28 gram/day). Two subsets of ESTHER participants were selected for DNA methylation assessment in the baseline blood samples: Subset I consists of 1,000 participants

consecutively enrolled during the first 3 months of recruitment; Subset II consists of 864 participants selected for a case-cohort design for mortality analysis [5]. The study was approved by the ethics committees of the University of Heidelberg and of the Medical Association of Saarland. All participants provided written informed consent.

KORA - This study is based on data from participants of four independent cross-sectional surveys (S1–S4) of the KORA (Cooperative Health Research in the Region of Augsburg) project between 1984 and 2001 [6], as well as from participants from KORA T2DM Family Study (T2DMFAM19 [7]), which was performed in 2001 / 2002. All probands were from the city or region of Augsburg. All participants were living in Germany and all were of European origin. DNA methylation was performed using the Illumina 450K BeadChip array. The highest educational attainment was categorized as follow: ‘low’ = primary school or lower; ‘medium’ = secondary school, ‘high’ = university degree or higher. Smoking was categorized as ‘never’, ‘former’ and ‘current’ smokers based on self-reported information. Alcohol was categorized as ‘abstainer’, ‘occasional’ (less than 28 g/day) and ‘habitual’ drinkers (more than 28 g/day). Height and weight were measured at enrolment with a standardized protocol, and body mass index (BMI) was calculated as the ratio between weight in kg and squared height in meters, treated as categorical variable: normal weight = BMI $\leq$ 25; overweight = 25 < BMI $\leq$ 30; obese = BMI $\geq$ 30.

MCCS - We used data from studies nested within the Melbourne Collaborative Cohort Study (Melbourne, Victoria, Australia), a prospective cohort study of 41,513 healthy adult volunteers (24,469 women) aged 27–76 years (99.3% were aged 40–69 years) at baseline between 1990 and 1994 (*MILNE, INT J EPIDEMIOL, 2017*). DNA samples used for the present analysis were extracted from peripheral blood drawn at the time of recruitment (1990–1994). For the majority (70%) of participants, the DNA source was dried blood spots collected onto Guthrie Card Diagnostic Cellulose filter paper (Whatman plc, Kent, United Kingdom) and stored in airtight containers at room temperature. The other sources of DNA were peripheral blood mononuclear cells and buffy coats stored at −80°C for 28% and 2% participants, respectively.

The study sample comprised Melbourne Collaborative Cohort Study participants selected as controls in nested case-control studies of breast, colorectal, kidney, lung, prostate, or urothelial cancer or mature B-cell malignancies [8-11]. Controls had been individually matched to cases on age (they had to be free of cancer at an age within 1 year of the age at diagnosis of the corresponding case), sex, country of birth, and blood DNA source (dried blood spot, peripheral blood mononuclear cells, or buffy coat). For all but the colorectal cancer study, controls were matched to cases on year of birth. For the lung cancer study, controls were matched on smoking status at the time of blood collection.

Socio-economic, dietary and lifestyle-related variables were collected at study enrolment through the use of a validated questionnaires. The highest educational attainment was categorized as follows: ‘low’ = primary school or lower; ‘medium’ = secondary school, ‘high’ = university degree or higher. Smoking was categorized as ‘never’, ‘former’ and ‘current’ smokers based on self-reported information. Alcohol was categorized as ‘abstainer’, ‘occasional’ (less than 28 g/day) and ‘habitual’ drinkers (more than 28 g/day). Height and weight were measured at enrolment with a standardized protocol, and body mass index (BMI) was calculated as the ratio between weight in kg and squared height in meters, treated as categorical variable: normal weight = BMI $\leq$ 25; overweight = 25 < BMI $\leq$ 30; obese = BMI $\geq$ 30. Physical activity was 1 to 4 and reflecting metabolic equivalents, as described in previous. Physical activity was defined by a summary score aimed to reflect the total energy expenditure as described in MacInnis et al., and was based on questions relating to frequency of walking, vigorous exercise (exercise ‘making you sweat or feel out of breath, and includes such activities as swimming, tennis, netball, athletics, and running’) and less vigorous exercise (exercise ‘which did not make you sweat or feel out of breath and includes such activities as bike riding, dancing, etc.’) over the last 6 months [12].

NAS - The Normative Aging Study (NAS) is an ongoing longitudinal male cohort established in 1963. Men were free of known chronic clinical conditions at enrolment and were subsequently invited to clinical examinations every 3 to 5 years [13]. At each visit, participants provided information on medical history, lifestyle, and demographic factors, and underwent a physical examination and laboratory tests. The NAS study was approved by the Institutional Review Boards (IRBs) of the participating institutions. Participants have provided written informed consent at each visit.

DNA samples were collected from 1999 to 2007 from the 675 active participants and used for DNA methylation analysis. We excluded participants who were not of European descent or had missing information on race, other covariates or with leukaemia or any blood cancer, leaving a total of 624 individuals for the analysis.

At each in-person examination visit, participants provide demographic information and completed a questionnaire enquiring about their smoking status, education, alcohol

consumption and life-style, including a measure of physical activity (metabolic equivalent of task: MET). Anthropometric measurements (height and weight) were also performed with participants in undershorts and socks. All variables were harmonized and categorized as in the EPIC study, except for smoking (ever/never), alcohol consumption (≤2 or >2 drinks/day), and physical activity (≤10 MET hours/week, 10<MET hours/week or >25 MET hours/week).

NICOLA – The Northern Ireland Cohort for the Longitudinal Study of Ageing is a longitudinal cohort representative of the non-institutionalized population of Northern Ireland over the age of 50 years (n=8,500) [14]. The study which was established in 2013 has three main components: a computer aided personal interview (CAPI), a self-completion questionnaire and health assessment. Dietary intake was also assessed by a food frequency questionnaire. The CAPI was extensive in scope and included assessment of demographic, social and health-related factors. Measures of cardiovascular, physical, cognitive and visual function were determined and a biobank of biological samples collected. Educational attainment was categorized as follows: 'low' = primary school or lower; 'medium' = secondary school, 'high' = higher education. Smoking was categorized as 'never', 'former' or 'current' based on self-reported information. Alcohol was categorized as 'abstainer', 'occasional' (on average less than one alcoholic beverage/day) and 'habitual' drinkers (on average one or more alcoholic beverages/day). Physical activity was defined as low, moderate or high based on the scoring protocol of the International Physical Activity Questionnaire (3). Physical activity was categorized as 'high' = highest tertile of metabolic equivalent (MET) computed based on self-reported frequency and duration of physical activity; 'medium' = middle tertile of MET; 'low' = lowest tertile of MET. Height and weight were measured at the health assessment with a standardized protocol, and body mass index (BMI) was calculated as the ratio between weight in kg and height in meters squared, treated as categorical variable: normal weight = BMI ≤ 25; overweight = 25 < BMI ≤ 30; obese = BMI ≥ 30.

Rotterdam Study - The Rotterdam Study is a prospective population based-study started in 1989. It is composed of residents of the neighborhood of Ommoord, Rotterdam, the Netherlands, aged 45 years and over. Data on socio-economic status, diet and lifestyle factors were assessed by standardized questionnaires, measured anthropometric data and blood samples collected for DNA extraction. Education was categorized into three groups: i) 'low' = primary school or lower; ii) 'medium' = secondary school; iii) 'high' = university degree or higher. Smoking was categorized as 'never', 'former' and 'current' smokers based on self-reported information. Physical activity level was measured with a self-administrated LASA Physical Activity questionnaire (LAPAQ). Later, the intensity of the reported activities was quantified using the metabolic equivalent of task (MET) hours per week, and then categorized in three groups: i) sedentary: <10 MET hours/week; ii) moderately active: 10–40 MET hours/week and iii) active: > 40 MET hours/week. Alcohol consumption was categorized into i) abstainer: consumption of 0 grams/day of alcohol, ii) moderate drinker: consumption > 0 grams/day and ≥ 28 grams/day, ii) habitual drinker: consumption > 28 grams/day. Height and weight were measured with a standardized protocol. Body mass index (BMI) was calculated as the ratio between weight in kg and squared height in meters, and then categorized in three groups: i) normal weight: BMI ≤ 25; ii) overweight: 25 < BMI ≤ 30; iii) obese = BMI ≥ 30

SAPALDIA - Swiss Study on Air Pollution and Lung and Heart Disease in Adults is a population cohort in Switzerland initiated in 1991 recruiting 9651 random samples from eight cities covering geographical, meteorological, and cultural diversity of the population. The SAPALDIA cohort has been described in detail previously [15]. Blood samples collected at the second follow-up in 2010-11 and stored at -80 °C. DNA methylation was analyzed in the framework of EXPOsOMICS for a total of 402 samples selected based on asthma status and the availability of archived blood samples and covariate information. Self-reported education level was categorized as 'high' = technical college or university; 'medium' = secondary school, middle school or apprenticeship; 'low' = primary school. Smoking status was categorized as 'never' or 'former' smokers based on self-reported information. Current smokers were excluded. Alcohol consumption was categorized as 'abstainer' = never; 'occasional' = rarely, 1-2 times per week, or several times per week; 'habitual' = once per day, twice per day, 3 times or more per day. Physical activity was categorized as 'high' = highest tertile of metabolic equivalent (MET) computed based on self-reported frequency and duration of physical activity; 'medium' = middle tertile of MET; 'low' = lowest tertile of MET. Vigorous physical activity was given 6 MET while moderate activity 3 MET. Weight and height were measured during the health examination. BMI was computed as weight in kilogram divided by squared height in meter and categorized as 'normal' = BMI < 25; 'overweight' = 25 ≤ BMI < 30; 'obese' = BMI ≥ 30.

SKIPOGH - The Swiss Kidney Project on Genes in Hypertension (SKIPOGH) study is a multicenter family-based population study initiated in 2009 to explore the genetic and environmental determinants of BP [16]. Study participants were recruited in the cantons of Bern and Geneva and the city of Lausanne. Recruitment began in

December 2009 and ended in April 2013. Inclusion criteria were: (i) written informed consent, (ii) minimum age of 18 years, (iii) Caucasian origin, and (iv) at least one, and preferably 3, first-degree family members also willing to participate. At the end of the recruitment period, the study population included 1,128 participants from 271 distinct family pedigrees. Of the individuals asked to participate in Bern, Geneva, and Lausanne, 21%, 22%, and 20% agreed, respectively. The SKIPOGH study was approved by the ethical committees of Lausanne University Hospital, Geneva University Hospital, and the University Hospital of Bern. Data were from the first follow-up which started in 2013, but highest attained education that was collected at baseline. Covariates in the analysis were categorised from self-report or clinical data as follows: Education was defined as low (no diploma or mandatory school or secondary vocational training), medium (secondary vocational training of superior level or superior non-university training) or high (university degree). Alcohol use was classed as non-drinker, occasional drinker (≤ 14 alcohol units/week for women and ≤ 21 alcohol units/week for men) or habitual drinker (> 14 alcohol units/week for women and >21 alcohol units/week for men). Physical activity was defined as low, moderate or high based on a question about overall physical activity: "Please indicate on a scale from 1-10 the physical efforts that you are doing on a daily basis, including those at work, during sports and your free time activities" [3]. Smoking was categorized as 'never', 'former' and 'current' smokers based on self-reported information. Height and weight were measured with a standardized protocol, and body mass index (BMI) was calculated as the ratio between weight in kg and squared height in meters, treated as categorical variable: normal weight = BMI ≤ 25; overweight = 25 < BMI ≤ 30; obese = BMI ≥ 30. An EDTA whole blood collection vessel was used (BD, Franklin Lakes, New Jersey). DNA was extracted using standard methods on a bead-based KingFisher Duo robot extraction system (ThermoFisher, Waltham, Massachusetts). DNA quality assessement and quantification was performed using a Nanodrop system (ThermoFisher, Waltham, Massachusetts). For bisulfite conversion, the protocol started with ~1.2ug of DNA extracted. For the PCR step: alternative incubation conditions was performed when using the Illumina Infinium® Methylation Assay (Appendix page 6 of bisulfite conversion protocol pdf). The final elution was done with 8ul of M-Elution Buffer. Processing pipeline of the beta methylation values was CPACOR [17].

TERRE: We used data from population-based controls of a case-control of Parkinson's disease in French farmers [18]. We included Parkinson's disease patients enrolled in the French health insurance system for farmers (MSA) from 62 metropolitan districts (1998-1999). We randomly selected eligible controls from among all MSA members who requested reimbursement for health expenses (participation rate = 83%). Controls were matched to cases on age, sex, and district of residency, and did not report cardinal signs of Parkinson's disease. DNA was extracted from peripheral blood leukocytes. Education was defined as low (no education or primary school), medium (certificate level) or high (secondary school to university degree). Alcohol use was classed as non-drinker, occasional drinker (e.g., events, family celebrations) or habitual drinker (regularly or daily drinker). Physical activity was not assessed. Smoking was categorized as 'never', 'former' and 'current' smokers based on self-reported information. Height and weight were self-reported, and body mass index (BMI) was calculated as the ratio between weight in kg and squared height in meters, treated as categorical variable: normal weight = BMI ≤ 25; overweight = 25 < BMI ≤ 30; obese = BMI ≥ 30.

The Irish Longitudinal Study on Ageing (TILDA) is a large prospective cohort study examining the social, economic and health circumstances of 8,175 community-dwelling older adults aged 50 years and over resident in the Republic of Ireland. The sample was generated using a 3-stage selection process and the Irish Geodirectory as the sampling frame. The Irish Geodirectory is a comprehensive listing of all addresses in the Republic of Ireland, which is compiled by the national post service and ordnance survey Ireland. Subdivisions of district electoral divisions pre-stratified by socio-economic status, age, and geographical location, served as the primary sampling units. The second stage involved the selection of a random sample of 40 addresses from within each PSU resulting in an initial sample of 25,600 addresses. The third stage involved the recruitment of all members of the household aged 50 years and over. Consequently, the response rate was defined as the proportion of households including an eligible participant from whom an interview was successfully obtained. A response rate of 62% was achieved at the household level. There were three components to the survey. Respondents completed a computer-assisted personal interview and a separate self-completion paper and pencil module which collected information that was considered sensitive. All participants were invited to undergo an independent health assessment at one of two national centers using trained nursing staff. Blood samples were taken during the clinical assessment with the consent of participants. A more detailed exposition of study design, sample selection and protocol is available elsewhere [19]. The present study sample included 500 healthy individuals: 125 for each of the four SES classes: stable professional, any downward mobility, any upward mobility, and stable unskilled (see socioeconomic position assessment). Buffy coat or peripheral blood mononuclear cells (PBMC) samples were available for all the individuals. Overall, after DNA

methylation data quality controls and sample filtering, 490 subjects were analyzed in this study.

Effects size comparison between SEMs and epigenetic clocks

For the three epigenetic clocks, the estimated differences presented in Table 2 (βs) represent the change in biological age (in years) compared with the reference group. In order to make the effect sizes of the logSEM variable comparable with those of the three epigenetic clocks (i.e. expressed as years of increasing biological age), we re-scaled both the effect sizes and the standard deviations by a factor $\sigma = \sigma_{EC} / \sigma_{SEMs}$, where σ_{EC} is the average standard deviation of the three epigenetic clocks and σ_{SEMs} is the standard deviation of the logSEM variable. In this way, the re-scaled effect size of logSEM can be interpreted as years of increasing biological age as is the case for the three epigenetic clocks.

SUPPLEMENTARY REFERENCES

1. Riboli E, Kaaks R. The EPIC Project: rationale and study design. European Prospective Investigation into Cancer and Nutrition. Int J Epidemiol. 1997 (Suppl 1); 26:S6–14. https://doi.org/10.1093/ije/26.suppl_1.S6
2. Elliott P, Vergnaud AC, Singh D, Neasham D, Spear J, Heard A. The Airwave Health Monitoring Study of police officers and staff in Great Britain: rationale, design and methods. Environ Res. 2014; 134:280–85. https://doi.org/10.1016/j.envres.2014.07.025
3. The IPAQ group. International Physical Activity Questionnaire. 2016. https://sites.google.com/site/theipaq/questionnaire_links
4. Raum E, Rothenbacher D, Löw M, Stegmaier C, Ziegler H, Brenner H. Changes of cardiovascular risk factors and their implications in subsequent birth cohorts of older adults in Germany: a life course approach. Eur J Cardiovasc Prev Rehabil. 2007; 14:809–14. https://doi.org/10.1097/HJR.0b013e3282eeb308
5. Zhang Y, Wilson R, Heiss J, Breitling LP, Saum KU, Schöttker B, Holleczek B, Waldenberger M, Peters A, Brenner H. DNA methylation signatures in peripheral blood strongly predict all-cause mortality. Nat Commun. 2017; 8:14617. https://doi.org/10.1038/ncomms14617
6. Wichmann HE, Gieger C, Illig T, and MONICA/KORA Study Group. KORA-gen--resource for population genetics, controls and a broad spectrum of disease phenotypes. Gesundheitswesen. 2005 (Suppl 1); 67:S26–30. https://doi.org/10.1055/s-2005-858226
7. Huth C, Illig T, Herder C, Gieger C, Grallert H, Vollmert C, Rathmann W, Hamid YH, Pedersen O, Hansen T, Thorand B, Meisinger C, Doring A, et al. Joint analysis of individual participants' data from 17 studies on the association of the IL6 variant -174G>C with circulating glucose levels, interleukin-6 levels, and body mass index. Ann Med. 2009; 41:128–38. https://doi.org/10.1080/07853890802337037
8. Severi G, Southey MC, English DR, Jung CH, Lonie A, McLean C, Tsimiklis H, Hopper JL, Giles GG, Baglietto L. Epigenome-wide methylation in DNA from peripheral blood as a marker of risk for breast cancer. Breast Cancer Res Treat. 2014; 148:665–73. https://doi.org/10.1007/s10549-014-3209-y
9. Dugué PA, Brinkman MT, Milne RL, Wong EM, FitzGerald LM, Bassett JK, Joo JE, Jung CH, Makalic E, Schmidt DF, Park DJ, Chung J, Ta AD, et al. Genome-wide measures of DNA methylation in peripheral blood and the risk of urothelial cell carcinoma: a prospective nested case-control study. Br J Cancer. 2016; 115:664–73. https://doi.org/10.1038/bjc.2016.237
10. Wong Doo N, Makalic E, Joo JE, Vajdic CM, Schmidt DF, Wong EM, Jung CH, Severi G, Park DJ, Chung J, Baglietto L, Prince HM, Seymour JF, et al. Global measures of peripheral blood-derived DNA methylation as a risk factor in the development of mature B-cell neoplasms. Epigenomics. 2016; 8:55–66. https://doi.org/10.2217/epi.15.97
11. Baglietto L, Ponzi E, Haycock P, Hodge A, Bianca Assumma M, Jung CH, Chung J, Fasanelli F, Guida F, Campanella G, Chadeau-Hyam M, Grankvist K, Johansson M, et al. DNA methylation changes measured in pre-diagnostic peripheral blood samples are associated with smoking and lung cancer risk. Int J Cancer. 2017; 140:50–61. https://doi.org/10.1002/ijc.30431
12. MacInnis RJ, English DR, Hopper JL, Haydon AM, Gertig DM, Giles GG. Body size and composition and colon cancer risk in men. Cancer Epidemiol Biomarkers Prev. 2004; 13:553–59.
13. Wilker E, Korrick S, Nie LH, Sparrow D, Vokonas P, Coull B, Wright RO, Schwartz J, Hu H. Longitudinal changes in bone lead levels: the VA Normative Aging Study. J Occup Environ Med. 2011; 53:850–55. https://doi.org/10.1097/JOM.0b013e31822589a9
14. Burns F, Carney GM, Cruise S, Devine P, Devlin A, Donnelly M, French D, Kee F, Montgomery L, O'Reilly D, Scott A, Tully MA. (2017). Early key findings from a study of older people in Northern Ireland. The NICOLA Study. (Belfast: Queen's University, Belfast).

15. Ackermann-Liebrich U, Kuna-Dibbert B, Probst-Hensch NM, Schindler C, Felber Dietrich D, Stutz EZ, Bayer-Oglesby L, Baum F, Brändli O, Brutsche M, Downs SH, Keidel D, Gerbase MW, et al, and SAPALDIA Team. Follow-up of the Swiss Cohort Study on Air Pollution and Lung Diseases in Adults (SAPALDIA 2) 1991-2003: methods and characterization of participants. Soz Praventivmed. 2005; 50:245–63. https://doi.org/10.1007/s00038-005-4075-5

16. Pruijm M, Ponte B, Ackermann D, Paccaud F, Guessous I, Ehret G, Pechère-Bertschi A, Vogt B, Mohaupt MG, Martin PY, Youhanna SC, Nägele N, Vollenweider P, et al. Associations of urinary uromodulin with clinical characteristics and markers of tubular function in the general population. Clin J Am Soc Nephrol. 2016; 11:70–80. https://doi.org/10.2215/CJN.04230415

17. Lehne B, Drong AW, Loh M, Zhang W, Scott WR, Tan ST, Afzal U, Scott J, Jarvelin MR, Elliott P, McCarthy MI, Kooner JS, Chambers JC. A coherent approach for analysis of the Illumina HumanMethylation450 BeadChip improves data quality and performance in epigenome-wide association studies. Genome Biol. 2015; 16:37. https://doi.org/10.1186/s13059-015-0600-x

18. Elbaz A, Clavel J, Rathouz PJ, Moisan F, Galanaud JP, Delemotte B, Alpérovitch A, Tzourio C. Professional exposure to pesticides and Parkinson disease. Ann Neurol. 2009; 66:494–504. https://doi.org/10.1002/ana.21717

19. Whelan BJ, Savva GM. Design and methodology of the Irish Longitudinal Study on Ageing. J Am Geriatr Soc. 2013 (Suppl 2); 61:S265–68. https://doi.org/10.1111/jgs.12199
