# Supplementary material for: Socioeconomic position, lifestyle habits and biomarkers of epigenetic aging: a multi-cohort analysis
Source: Aging (Albany NY). 2019 Apr 14;11(7):2045–70. doi: 10.18632/aging.101900 (PMC6503871; doi:10.18632/aging.101900)
Supplement: Supplementary Tables [file aging-11-101900-s002.pdf]

SUPPLEMENTARY TABLES

Table S1. Results of linear regressions using epigenetic aging biomarkers (WBC adjusted) as outcomes and lifestyle related risk factors as predictors.

		SEMs		HorvathEAA	
		Model 1	Model 2	Model 1	Model 2
Education (ref: High)	Medium	**0.28 (0.06; 0.49)***	**0.23 (-0.02; 0.48)**$^{+}$	0.1 (-0.08; 0.28)	0.09 (-0.1; 0.29)
	High	**0.32 (0.09; 0.54)****	**0.27 (0.04; 0.5)***	**0.19 (-0.01; 0.38)**$^{+}$	0.14 (-0.06; 0.34)
Smoking (ref: Never)	Former	**0.24 (0.08; 0.4)****	**0.28 (0.12; 0.43)*****	**0.18 (0.02; 0.35)***	**0.17 (0.01; 0.32)***
	Current	**0.54 (0.32; 0.76)*****	**0.54 (0.32; 0.77)*****	0.12 (-0.07; 0.31)	0.1 (-0.1; 0.3)
Obesity (ref: BMI < 25)	BMI < 30	0.02 (-0.17; 0.2)	0 (-0.18; 0.17)	**0.37 (0.22; 0.52)*****	**0.35 (0.19; 0.5)*****
	BMI ≥ 30	-0.11 (-0.33; 0.1)	-0.13 (-0.34; 0.08)	**0.45 (0.27; 0.63)*****	**0.44 (0.25; 0.62)*****
Alcohol (ref: Abstainer)	Occasional	**-0.16 (-0.35; 0.03)**$^{+}$	-0.14 (-0.33; 0.04)	-0.01 (-0.19; 0.16)	0.02 (-0.16; 0.19)
	Habitual	0.18 (-0.06; 0.42)	0.13 (-0.12; 0.38)	**0.2 (-0.03; 0.44)**$^{+}$	**0.26 (0.03; 0.49)***
Physical activity (ref: High)	Medium	-0.04 (-0.26; 0.18)	-0.06 (-0.24; 0.13)	0.08 (-0.08; 0.24)	0.07 (-0.09; 0.24)
	Low	0.03 (-0.27; 0.33)	-0.02 (-0.3; 0.26)	**0.22 (0.05; 0.4)***	**0.19 (0.01; 0.37)***

		HannumEAA		LevineEAA	
		Model 1	Model 2	Model 1	Model 2
Education (ref: High)	Medium	**0.3 (0.14; 0.46)*****	**0.24 (0.06; 0.42)****	**0.36 (0.05; 0.68)***	0.16 (-0.23; 0.55)
	High	**0.32 (0.16; 0.48)*****	**0.28 (0.12; 0.43)*****	**0.85 (0.53; 1.17)*****	**0.57 (0.25; 0.89)*****
Smoking (ref: Never)	Former	**0.15 (0.03; 0.26)***	**0.1 (-0.01; 0.22)**$^{+}$	**0.68 (0.49; 0.87)*****	**0.58 (0.38; 0.78)*****
	Current	**0.46 (0.26; 0.65)*****	**0.4 (0.21; 0.6)*****	**1.62 (1.35; 1.89)*****	**1.48 (1.19; 1.77)*****
Obesity (ref: BMI < 25)	BMI < 30	**0.2 (0.08; 0.32)****	**0.19 (0.06; 0.32)****	**0.59 (0.3; 0.88)*****	**0.5 (0.26; 0.74)*****
	BMI ≥ 30	**0.25 (0.11; 0.39)*****	**0.24 (0.1; 0.39)*****	**1.25 (0.92; 1.58)*****	**1.18 (0.87; 1.48)*****
Alcohol (ref: Abstainer)	Occasional	-0.02 (-0.15; 0.11)	0.05 (-0.09; 0.18)	-0.07 (-0.33; 0.19)	0.09 (-0.14; 0.32)
	Habitual	**0.24 (0.06; 0.42)****	**0.3 (0.1; 0.49)****	**0.91 (0.61; 1.2)*****	**0.99 (0.66; 1.31)*****
Physical activity (ref: High)	Medium	0.09 (-0.07; 0.24)	0.07 (-0.08; 0.22)	0.17 (-0.17; 0.52)	0.12 (-0.13; 0.37)
	Low	0.13 (-0.11; 0.37)	0.07 (-0.19; 0.33)	0.49 (-0.01; 0.98)$^{+}$	0.27 (-0.13; 0.67)

***** p < 0.001; ** p < 0.01; * p < 0.05; + p < 0.10**

Model 1 includes age, sex, and cohort specific covariates; Model 2 includes additional adjustment for education, smoking, BMI, alcohol and physical activity.

Table S2. Interaction with age and sex.

		SEMs		HorvathEAA	
		age	sex	age	sex
**Education (ref: High)**	Medium	0.02 (-0.01; 0.05)	**-0.08 (-0.16; 0)**$^{+}$	0 (-0.03; 0.03)	0.02 (-0.39; 0.43)
	High	0.03 (0; 0.05)$^{+}$	-0.05 (-0.13; 0.02)	-0.01 (-0.04; 0.02)	0.19 (-0.22; 0.59)
**Smoking (ref: Never)**	Former	0.01 (0; 0.03)	-0.07 (-0.15; 0)$^{+}$	**0.02 (0; 0.04)***	0 (-0.4; 0.4)
	Current	**0.04 (0.01; 0.06)****	-0.06 (-0.14; 0.02)	**0.03 (0.01; 0.05)***	0.21 (-0.19; 0.61)
**Obesity (ref: BMI < 25)**	BMI < 30	**0.03 (0.01; 0.06)***	-0.06 (-0.14; 0.02)	0 (-0.03; 0.03)	0.03 (-0.37; 0.43)
	BMI ≥ 30	**0.02 (0; 0.04)**$^{+}$	-0.05 (-0.13; 0.02)	0 (-0.02; 0.02)	0.23 (-0.17; 0.63)
**Alcohol (ref: Abstainer)**	Occasional	-0.01 (-0.04; 0.01)	-0.08 (-0.15; 0)$^{+}$	-0.01 (-0.03; 0.02)	0.01 (-0.39; 0.41)
	Habitual	0.01 (-0.01; 0.04)	-0.06 (-0.14; 0.02)	0 (-0.02; 0.03)	0.19 (-0.21; 0.59)
**Physical activity (ref: High)**	Medium	0 (-0.03; 0.02)	-0.08 (-0.16; 0)$^{+}$	-0.02 (-0.04; 0)	0 (-0.4; 0.4)
	Low	0 (-0.02; 0.03)	-0.06 (-0.14; 0.02)	-0.01 (-0.03; 0.02)	0.16 (-0.24; 0.57)

		HannumEAA		LevineEAA	
		Age	Sex	Age	sex
**Education (ref: High)**	Medium	0.01 (-0.01; 0.02)	0.03 (-0.32; 0.38)	0 (-0.04; 0.03)	0.13 (-0.44; 0.7)
	High	**-0.02 (-0.04; 0)***	0.1 (-0.22; 0.43)	-0.01 (-0.05; 0.02)	-0.08 (-0.74; 0.59)
**Smoking (ref: Never)**	Former	**0.01 (0; 0.03)***	-0.01 (-0.37; 0.35)	**0.02 (0; 0.04)**$^{+}$	0.09 (-0.47; 0.65)
	Current	**0.04 (0.02; 0.06)*****	0.11 (-0.21; 0.43)	**0.03 (0; 0.07)**$^{+}$	-0.06 (-0.72; 0.6)
**Obesity (ref: BMI < 25)**	BMI < 30	-0.01 (-0.03; 0.02)	0.05 (-0.29; 0.4)	0 (-0.03; 0.04)	0.11 (-0.45; 0.67)
	BMI ≥ 30	-0.01 (-0.02; 0.01)	0.13 (-0.19; 0.46)	0 (-0.03; 0.03)	-0.04 (-0.7; 0.63)
**Alcohol (ref: Abstainer)**	Occasional	0 (-0.02; 0.02)	0.03 (-0.31; 0.37)	0.01 (-0.02; 0.04)	0.08 (-0.48; 0.64)
	Habitual	0 (-0.02; 0.02)	0.1 (-0.22; 0.43)	0.01 (-0.02; 0.05)	-0.08 (-0.75; 0.58)
**Physical activity (ref: High)**	Medium	0 (-0.02; 0.01)	0 (-0.35; 0.35)	**-0.03 (-0.06; 0)**$^{+}$	0.12 (-0.44; 0.68)
	Low	0 (-0.02; 0.02)	0.07 (-0.25; 0.4)	0 (-0.04; 0.03)	-0.07 (-0.73; 0.59)

***** p < 0.001; ** p < 0.01; * p < 0.05; + p < 0.10**

Table S3. Results of the enrichment analyses using the classification of the UCSC Genome Browser for the relationship with CpG islands, open chromatin state and DNase hypersensitivity.

Relation to CpG island according to UCSC Genome Browser	Permutation based p-value for enrichment
Shores	0.02
Open chromatin evidence	0.03
CpG Island	0.27
Shelves	1
Non CpG Island	1
DNase hypersensitivity evidence	1

P-values were computed according to the algorithm implemented in the *regioneR* R package.

Table S4. Results of the enrichment analyses using the ENCODE classification for chromatin states in embryonic stem cell (H1-hESC).

Chromatin state according to ENCODE ChIP-Seq	Permutation based p-value for enrichment
Heterochromatin / Low signal / CNV	< 0.0001
Inactive / Poised promoter	< 0.0001
Polycomb Repressed	0.001
Transcriptional elongation / Transition	1
Weak Transcribed	1
Active promoter	1
Weak promoter	1
Strong enhancer	1
Weak / Poised enhancer	1
Insulator	1
Non regulatory elements	1

P-values were computed according to the algorithm implemented in the *regioneR* R package.

Table S5. Results of the enrichment analyses using the ENCODE classification for 58 protein TFBS in non-treated embryonic stem cell (H1-hESC).

Transcription Factor	Lab	Permutation based p-value for enrichment
EZH2	Broad	< 0.0001
SUZ12	USC	< 0.0001
CtBP2	USC	0.15
CHD1	Broad	0.27
NRSF	HudsonAlpha	0.88
BCL11A	HudsonAlpha	1
c-Myc	UT-A	1
POU5F1	HudsonAlpha	1
MafK	Stanford	1
RXRA	HudsonAlpha	1
NANOG	HudsonAlpha	1
CHD1	Stanford	1
c-Jun	Stanford	1
RFX5	Stanford	1
HDAC2	HudsonAlpha	1
FOSL1	HudsonAlpha	1
TCF12	Stanford	1
CEBPB	HudsonAlpha	1
TEAD4	USC	1
Max	UT-A	1
CTCF	Stanford	1
Rad21	Stanford	1
Rad21	HudsonAlpha	1
BRCA1	Stanford	1
CTCF	Broad	1
CTCF	Stanford	1
USF2	Stanford	1
SP2	HudsonAlpha	1
JARID1A	Broad	1
SIX5	HudsonAlpha	1
SRF	HudsonAlpha	1
USF-1	HudsonAlpha	1
ATF2	HudsonAlpha	1
JunD	Stanford	1
ATF3	HudsonAlpha	1
Bach1	Stanford	1
Nrf1	Stanford	1
c-Myc	Stanford	1

JunD	HudsonAlpha	1
GABP	HudsonAlpha	1
GTF2F1	Stanford	1
p300	HudsonAlpha	1
Egr-1	HudsonAlpha	1
Mxi1	Stanford	1
CHD2	Stanford	1
Znf143	Stanford	1
Sin3Ak-20	HudsonAlpha	1
RBBP5	Broad	1
YY1	HudsonAlpha	1
SP1	HudsonAlpha	1
SP4	HudsonAlpha	1
TAF7	HudsonAlpha	1
TBP	Stanford	1
Pol2	HudsonAlpha	1
SIN3A	Stanford	1
Pol2	UT-A	1
TAF1	HudsonAlpha	1
Pol2	HudsonAlpha	1

P-values were computed according to the algorithm implemented in the *regioneR* R package.
